# Effect of Humidity and Hydrophobicity on the Tribological Properties of Self-Assembled Monolayers

**DOI:** 10.1155/2013/748295

**Published:** 2013-05-02

**Authors:** Yen-Chih Liao, William Hargrove, Brandon L. Weeks

**Affiliations:** Department of Chemical Engineering, Texas Tech University, 6th Street, Canton, Lubbock, TX 79409, USA

## Abstract

The tribological properties of two distinctive alkanethiol SAMs, 16-mercaptohexadecanoic acid (MHA) and 1-octadecanethiol (ODT), on gold substrates in various humidity conditions were examined by lateral force microscopy (LFM). The results suggest that hydrophobic ODT SAM is insensitive to humidity. The difference of lateral force signal is within ±10% regardless of humidity. The lateral force signal of hydrophilic MHA SAMs has a significant decrease in signal in humid environments. The influence of bulk water was also investigated by LFM. By imaging under water, the capillary force is eliminated on ODT SAMs, which leads to a lower lateral force. However, the lateral force image was reversed on MHA SAMs, which suggested that hydrophobic forces dominated in water.

## 1. Introduction

Tribology is one of the most important mechanical properties for the interaction of surfaces. With the development of micro- and nanotechnology, the need for understanding tribological characteristic in micro- and nanoscale systems is important. Microelectromechanical systems (MEMS) have grown dramatically in fundamental and applied research in the past several decades [1–3]. MEMS devices have a large working surface area compared to regular mechanical devices. Thus, the tribological properties, including friction, adhesion, and wear, in MEMS are more critical than in bulk devices [4]. In order to effectively reduce the friction in the contact boundary, lubricants in micro/nanoscale are of significant interest to tribology research. Since the surface separation of a MEMS device is typically less than 5 *μ*m, ultrathin films with high packing density have become popular lubricant materials. A common material used is self-assembled monolayers (SAMs) due to their advantages of simple preparation, well-defined structure, and high sustainability in various environmental conditions. It is because of these characteristics that SAMs have the potential of being an ideal boundary protection layer and lubricant in MEMS. Among different types of SAM coating, alkanesilane molecules form rigid chemical bonds and well-ordered monolayers are formed on hydroxyl terminated surfaces and alkanethiol molecules on noble metal substrates.

Many studies have reported the tribological properties of SAMs [5–18], including the influence of SAMs' structure, the effects of terminal functional group, and the difference in various environmental conditions. The majority of the literature has focused on the humidity effect of alkysilane SAMs [7, 9–14, 16]. However, the study of humidity and terminal group influence on the friction properties of alkanethiol SAMs lacks many details.

Li et al. [8] performed the first study on the humidity effect on the frictional properties of mixed alkanethiol SAMs system with scanning force microscopy (SFM). By controlling the composition of two different alkanethiols, dodecanethiol and 11-mercapto-1-undecanol, the surface properties can vary from hydrophobic to hydrophilic. They also found that the composition of two different alkanethiols is sensitive to the friction coefficient only at lower humidity (below 50%). Gojzewski et al. [17] used dynamic force spectroscopy to examine the adhesion force of different functional group and chain length alkanethiols under various humidity. In their report, hydrophilic alkanethiols (–OH terminated) appear to have a higher adhesion force than hydrophobic alkanethiols (CH_3_ terminated) at all humidity conditions. Furthermore, the magnitude of adhesion force reached the highest value under a relative humidity between 40% and 80% for both hydrophobic and hydrophilic alkanethiols. Ahn et al. [18] did a comprehensive study of the influence of tribological properties on the alkanethiols with various functional groups and structures. They discovered that carboxyl- (–COOH-) terminated alkanethiols have the highest friction coefficient and surface energy compared to hydroxyl- (–OH-) terminated alkanethiols and alkyl- (–CH_3_-) terminated alkanethiols.

The goal of this study is to understand how relative humidity influences the tribological properties on a single composition of hydrophobic and hydrophilic alkanethiols SAMs with designed patterns on the substrates. The comparison of the friction properties difference between the alkanethiols and the substrates is also concluded. The experiments are conducted by lateral force microscopy (LFM) with the relative humidity from 0% to 60%, as well as under water.

## 2. Materials and Methods

### 2.1. Materials

Two alkanethiol chemicals were used as model materials: 16-mercaptohexadecanoic acid (MHA, HS(CH_2_)_15_COOH, 90%, Sigma-Aldrich Inc., St. Louis, MO, USA) as the hydrophilic thiol and 1-octadecanethiol (ODT, HS(CH_2_)_17_CH_3_, 98%, Sigma-Aldrich Inc., St. Louis, MO, USA) as the hydrophobic thiol. Absolute ethanol (99.99%, Sigma-Aldrich Inc., St. Louis, MO, USA) was used as the solvent and cleaning agent. Gold films were prepared by coating thermal evaporation of 100 nm of gold (99.99%, VWR International, LLC, Radnor, PA, USA) onto cleaved mica (Allied Electronics Inc., Fort Worth, TX, USA) at 10^−6^–10^−7^ torr [19]. Glass slides (plain, precleaned, 1.0 mm thick, VWR international, LLC, Radnor, PA, USA) were diced into 0.5 cm square pieces and cleaned with piranha solution containing H_2_O_2_ (30% solution) and H_2_SO_4_ (16 M) by ratio of 1 : 3 for 20 minutes [20]. The glass was rinsed with deionized water after cleaning. The gold substrates were made by a template stripping method [21] by gluing the glass pieces onto the gold film of the coated mica using Epo-Tek 377 (Epoxy Technology Inc., Billerica, NM, USA) and annealed at 100–150°C for two hours. The gold substrates were stored in vacuum desiccators until needed. The glass was peeled from the mica before use, leaving a fresh gold surface for microcontact printing (*μ*-CP).

### 2.2. Preparation of SAMs Film

In the *μ*-CP procedure, microstructures were transferred from a pattern-designed mask onto a polydimethylsiloxane (PDMS) stamp. The stamps in this study were provided by Lawrence Livermore National Laboratory. The concentration of the MHA and ODT solutions was 10 mM in absolute ethanol. The solution was added dropwise to the PDMS stamp until the stamp was completely covered by the solution and dried in air prior to use. The PDMS stamp was then physically brought to contact with the fresh gold surface for 12 hours. Details of the printing process and fabrication methods used to prepare stamps are described elsewhere [22]. 

### 2.3. Surface Properties Test and Humidity Control

The contact angles of the alkanethiol SAMs and fresh cleaved gold substrate in this study were evaluated by adding a droplet of deionized water (18 MΩ) on the surfaces at 20°C in a goniometer. Each material was tested several times until the error of the contact angle was within 3%.

Friction forces of MHA and ODT SAM-coated gold substrates were measured by AFM (NanoScope MultiMode IIIa Bruker Corporation Inc., Santa Barbara, CA, USA) in the LFM mode. Triangular silicon nitride (Si_3_N_4_) cantilevers were used in this research. The same probe was continuously used throughout the entire experiment in order to reduce variables in spring constant between each scan. Both trace and retrace directions were recorded in each scan. The lateral force signal for each scan was determined by subtracting trace and retrace lateral force signal measurements (subtracting the forward from the reverse signal) [23]. Furthermore, by averaging the values obtained from at least 5 experiment sets under the same conditions, the relative lateral force signal was determined.

Relative humidity (RH) of the AFM scanning environment was controlled using a home-built humidity chamber. The humidity chamber was designed on the basis of the principle of mixing dry air (~0%) and wet air (80%). Two air sources were continuously flowing into the mixing chamber. By controlling the flow rate of dry air and moisture-saturated air, the RH of the air pumped into the chamber can be controlled between the value of ~0% and 80% and maintained contact within 5%. In order to let humid air flow through the whole chamber, the inlet air enters from the top and leaves from the bottom. The humidity chamber encloses the scanning parts of the AFM, including the scanner, tip assembly, optical lever detection system, sample, and the side-mounted optical microscope. RH in the chamber was monitored by a hydrometer (Thermo Fisher Scientific Inc., Waltham, MA, USA) placed in the center. The RH in the humidity chamber took approximately 30 minutes to attain equilibrium, before each AFM measurement was conducted. The lateral force signal at 30 minutes scanning was considered as the equilibrium condition value and was used as the reference in the normalization process of lateral force signal.

## 3. Results and Discussion

In this study, two distinctive alkanethiols were used as the representative SAMs for hydrophobic and hydrophilic surfaces. In order to quantify the hydrophobicity of both alkanethiols, the magnitudes of the contact angles were evaluated by goniometer as shown in Table 1. The contact angle of freshly cleaved gold from template strip method is 72°. According to the measurement, gold substrate is a hydrophilic surface. MHA has a lower contact angle, 42°, which indicates its high wettability on the surface. ODT SAM has the hydrophobic surface with contact angle of 102°. Therefore, it was determined that the hydrophobicity of these surfaces from most to least hydrophobic were ODT, gold, and MHA, respectively.

**Figure 1:**
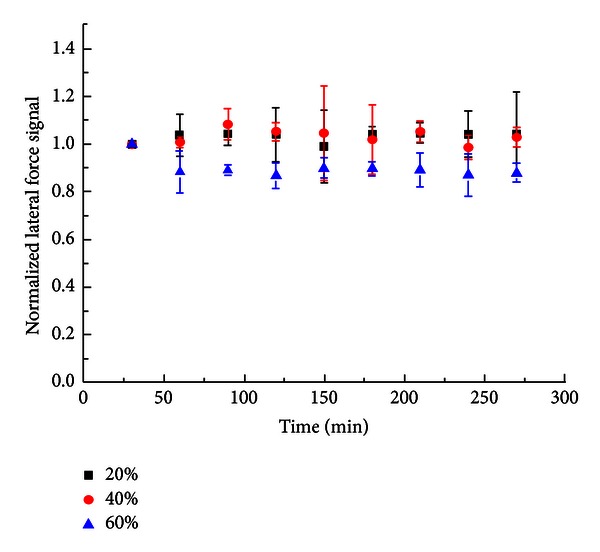
Normalized lateral force signal of ODT at different relative humidity conditions.

Figure 1 shows the time-dependent lateral force change of ODT SAM under different relative humidity conditions from 20% to 60%. The figure demonstrates that the lateral force changes for RH of 20% and 40% are nearly identical, and the magnitude of lateral force remains about the same as the equilibrium condition. At 60% RH, the lateral force decreases by 10% of its equilibrium condition value after 60 minutes. Then it remains a steady lateral force signal value afterward. But overall, the lateral force of ODT SAMs in various relative humidity conditions is stable throughout the entire experiment.

Due to the hydrophobicity of alkyl SAMs, simulation work done by Lorenz et al. [16] of the water layer on alkylsilane SAMs surface indicates that the presence of water causes the air-induced repulsive region to be softer than it is under dry condition. Thus, a lower friction coefficient could be expected on alkylsilane surface. Experimental results also discovered similar reduction of the friction properties on alkylsilane SAMs by AFM [11, 15]. They proposed that the water molecules on alkylsilane surface would cause molecular defect generation in the SAMs' structure, as well as the penetration of water into SAMs' free volume. However, the friction force response on alkylthiol is different from an alkylsilane even though they have similar contact condition on the water-SAM interfaces. Qian et al. [15] suggested that there is no hole for water molecules to penetrate through the monolayer due to the definite registry of the alkylthiol molecules on the gold substrate. In other words, alkylthiol SAMs are insensitive to the humidity. Our experimental results support the hypothesis in Qian's study. For all the humidity condition in this study, the lateral force signal did not have a significant change (within ±10%) after applying humid air into the alkylthiol SAMs surface. Sirghi [24] also proposed that the effect of humidity on the friction force on hydrophobic surface (contact angle of 100°) is weak. However, the sequence of the lateral force signal in different RH has coincident trend to the friction coefficient in Khatri et al.'s study [11]. 

**Figure 2:**
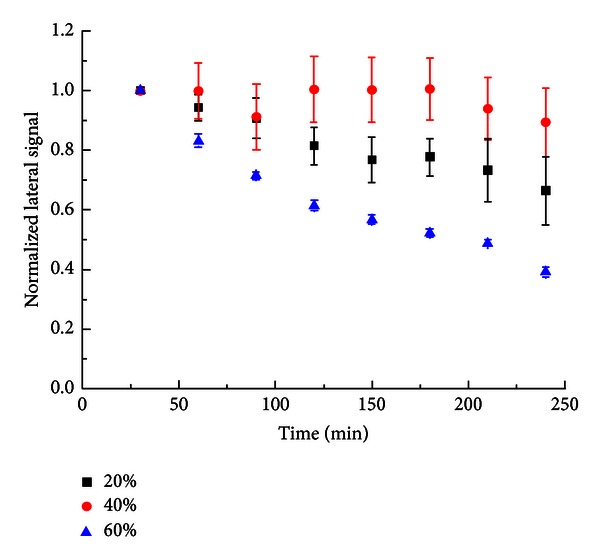
Normalized lateral force signal of MHA at different relative humidity conditions.

Different from ODT SAM, Figure 2 shows a distinctive time-dependent lateral force change of MHA SAM under different relative humidity conditions from 20% to 60%. Generally, the lateral force of MHA SAMs has significant decrease in the humid environments, especially 20% and 60%. The decreasing trend of the lateral force signal on 20% and 60% is similar, which the lateral force signal gradually declines the magnitude throughout the time. However, the amount of lateral force change at 60% RH is greater than at 20% RH.

The decreasing trend of lateral force signal in all RH indicated that the aggregate water in the tip-SAMs interface significantly reduce the surface friction force. The hydrophilic SAM surface does not have a repulsive region in between the water layer and the carboxyl end of the SAM. Thus, when the humid air (RH > 0%) enters the system, the water molecules will form a layer directly on the surface of the SAM over time. The thickness of the water will depend on the relative humidity and time. The longer the MHA SAMs were held in the humid condition, the lower lateral force could be observed. Li et al. [8] discovered that the friction coefficient of hydrophilic SAMs surface has significant reduction from 10% RH to 50% RH, which is coherent to our results. They suggested the friction properties dependent on the chain length and the size and the chemical nature of terminal group of the SAMs. At low humidity (20% RH), water molecules build up a thin layer at lower speed on the SAMs surface. The lateral force signal gradually decreases over time. The lateral force response at 40% RH also shows a decreasing trend in general. However, the fluctuation of the lateral force signal at 40% RH is still unclear. The data set of 40% RH is not significantly different than the 20% RH from statistical analysis at the 95% confidence level.

**Figure 3:**
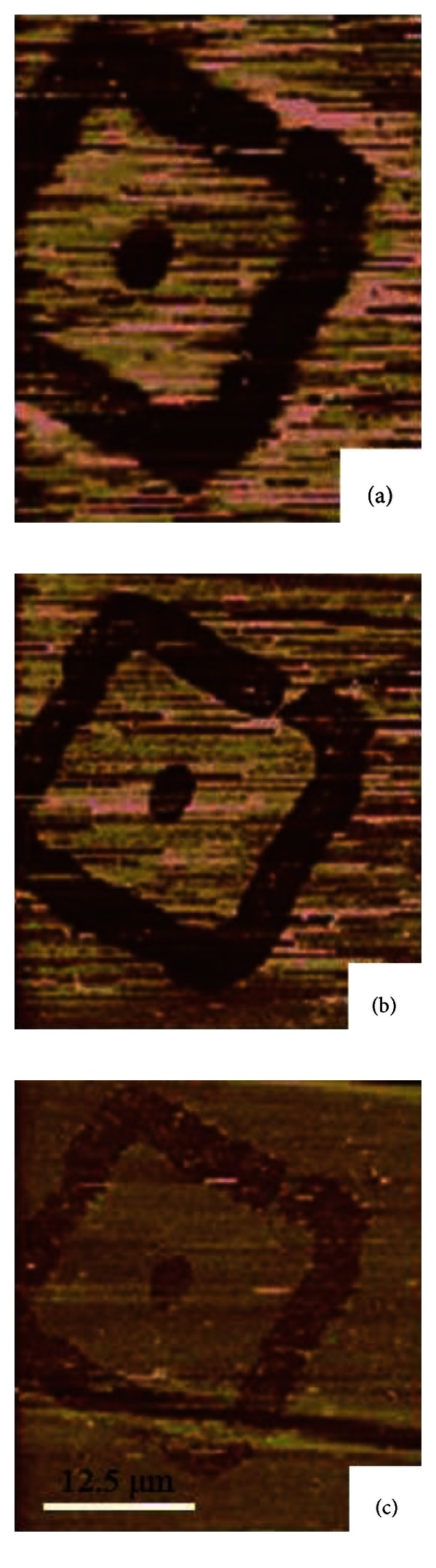
The lateral force images of ODT SAMs in (a) air (20% RH), (b) water for 5 minutes, and (c) water for 70 minutes. The dark part is the ODT SAM pattern, and the bright part is the gold substrate. The scale bar in (c) is the same for all images.

Unlike investigating the tribological properties on a bulk SAMs in other studies, a distinctive pattern of SAMs on the substrates was used in our work. Due to the design of the pattern, there are two boundary systems for the alkanethiols SAMs on the gold substrates: hydrophilic-hydrophobic (gold-ODT SAM) and hydrophilic-hydrophilic (gold-MHA SAM). In order to better understand the influence of water on lateral force signal in different boundary conditions, the lateral force images were scanned in the water phase. The lateral force images of ODT SAMs in different environment are as shown in Figure 3. The images indicate that the lateral force signal has significant decrease after water covers the ODT SAMs surface, which shows to be similar to the previous result at high humidity. It is obvious that the effect of capillary was eliminated in the water phase for hydrophobic surface in microscale [25]. The image also shows that hydrophobic-hydrophilic (gold-ODT SAMs) boundary is more clear in water than in air. The pattern of the image in air is larger than in water. It could be considered as the nature of the surface hydrophobicity. The hydrophobicity is more critical in the water phase and differentiates the boundary of two distinct surfaces. Thus, a well-defined boundary between gold and ODT SAMs could be observed.

**Figure 4:**
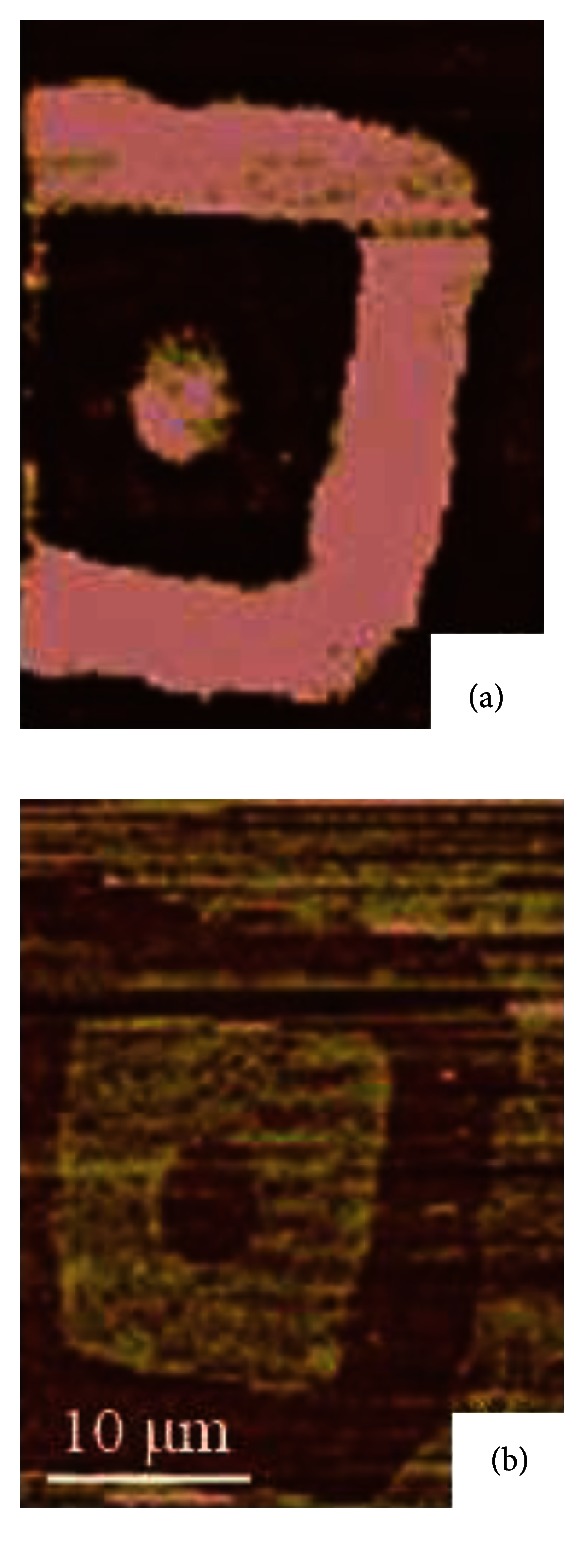
The lateral force images of MHA SAMs in (a) air (20% RH) and (b) water. The square pattern is the MHA SAM pattern, and the rest of the part is the gold substrate. The scale bar in (b) is the same for both images.

The MHA SAMs have a disparate lateral force response in water as shown in Figure 4. As expected, the lateral force of MHA SAMs decreases while scanning in water. However, the contrast of SAM patterns and gold substrates is reversed, which indicates that gold substrate has higher lateral force than the MHA SAMs in water. Vezenov et al. [25] proposed that the contrast of the lateral force image in water reflects the dominant effect of the hydrophobic force. Thus, other chemical interactions would be minimized. In the hydrophilic-hydrophilic (gold-MHA SAMs) system in this study, gold has higher hydrophobicity than MHA, which results in a light color. Compared to the ODT SAMs, the interfacial boundary on MHA SAMs was not emphasized by water. 

## 4. Conclusions

The tribological properties of hydrophobic and hydrophilic alkanethiol SAMs on gold substrates under various humidity conditions were examined by lateral force microscopy (LFM) in this study. For hydrophobic ODT SAMs, the lateral force signal was not strongly affected by humid environments. The magnitude of the lateral force signal slightly increases by 10% and 5% at 20% RH and 40% RH, respectively, but decreases by 10% at 60% RH. The decreasing of lateral force signal at high humidity might be contributed by aggregating sufficient water on the surface of alkylthiol SAMs. The fast-built water layer softens the repulsive region between the tip-alkylthiol interfaces and results in a lower lateral force signal. For hydrophilic MHA SAMs, the lateral force was significantly reduced with respect to RH. The presence of water layer can be considered as the lubricant layer on the hydrophilic SAMs surface. The decreasing trends of the lateral force signal on MHA SAMs in different humidity conditions are similar, which indicates that the lateral force signal depends more on the time spent in the humid environment than the humidity condition. 

Both ODT and MHA SAMs were successfully scanned by LFM in water to investigate the boundary and hydrophobicity effect. The capillary force in hydrophilic-hydrophobic gold-ODT SAMs system was eliminated by water and led to a lower contrast and magnitude on lateral force signal. The boundary between gold substrate and ODT SAMs was also minimized in water environment. A reversed contrast lateral force image of MHA SAMs in water could be observed. The hydrophobic force in water is more dominant, which obstructed other chemical interactions on the surface while scanning. Thus, a reversed contrast lateral force image was observed in the water. The water did not have an apparent influence on the hydrophilic-hydrophilic gold-ODT SAMs boundary.

## Figures and Tables

**Table 1:** Contact angles of water on different surfaces.

	Contact angle (°)
Bare gold surface	72
1-octadecanethiol (ODT)	102
16-mercaptohexadecanoic acid (MHA)	42
